# An algorithm for calculating exam quality as a basis for performance-based allocation of funds at medical schools

**DOI:** 10.3205/zma001043

**Published:** 2016-05-17

**Authors:** Timo Kirschstein, Alexander Wolters, Jan-Hendrik Lenz, Susanne Fröhlich, Oliver Hakenberg, Günther Kundt, Martin Darmüntzel, Michael Hecker, Attila Altiner, Brigitte Müller-Hilke

**Affiliations:** 1 Universitätsmedizin Rostock, "core group" zur Verbesserung der Lehre, Rostock, Deutschland; 2 Universitätsmedizin Rostock, Institut für Biostatistik und Informatik in Medizin und Alternsforschung, Rostock, Deutschland; 3 Universitätsmedizin Rostock, Studiendekanat, Rostock, Deutschland; 4 Universitätsmedizin Rostock, Klinik und Poliklinik für Neurologie, Zentrum für Nervenheilkunde, Rostock, Deutschland; 5 Universitätsmedizin Rostock, Institut für Immunologie, Rostock, Deutschland

**Keywords:** exam quality, item difficulty, discrimination, reliability, performance based allocation of funds in teaching

## Abstract

**Objective: **The amendment of the Medical Licensing Act (ÄAppO) in Germany in 2002 led to the introduction of graded assessments in the clinical part of medical studies. This, in turn, lent new weight to the importance of written tests, even though the minimum requirements for exam quality are sometimes difficult to reach. Introducing exam quality as a criterion for the award of performance-based allocation of funds is expected to steer the attention of faculty members towards more quality and perpetuate higher standards. However, at present there is a lack of suitable algorithms for calculating exam quality.

**Methods:** In the spring of 2014, the students‘ dean commissioned the „core group“ for curricular improvement at the University Medical Center in Rostock to revise the criteria for the allocation of performance-based funds for teaching. In a first approach, we developed an algorithm that was based on the results of the most common type of exam in medical education, multiple choice tests. It included item difficulty and discrimination, reliability as well as the distribution of grades achieved.

**Results: **This algorithm quantitatively describes exam quality of multiple choice exams. However, it can also be applied to exams involving short assay questions and the OSCE. It thus allows for the quantitation of exam quality in the various subjects and – in analogy to impact factors and third party grants – a ranking among faculty.

**Conclusion: **Our algorithm can be applied to all test formats in which item difficulty, the discriminatory power of the individual items, reliability of the exam and the distribution of grades are measured. Even though the content validity of an exam is not considered here, we believe that our algorithm is suitable as a general basis for performance-based allocation of funds.

## Zusammenfassung

**Zielsetzung:** Mit der Novellierung der ÄAppO im Jahr 2002 und der Einführung benoteter Leistungsnachweise hat die Bedeutung schriftlicher Prüfungen im klinischen Abschnitt des Medizinstudiums deutlich zugenommen. Allerdings werden die Mindestanforderungen an die Qualität von Prüfungen mitunter nur schwer erreicht. Mit der Aufnahme von Prüfungsqualität in den Kriterienkatalog zur Vergabe von LOM (Leistungsorientierter Mittelvergabe) könnte die Aufmerksamkeit der Lehrenden gelenkt und allein dadurch die Qualität der Prüfungen verbessert und verstetigt werden. Derzeit mangelt es jedoch an geeigneten Bemessungsgrundlagen.

**Methoden:** Im Frühjahr 2014 wurde die „core group“ zur Verbesserung der Lehre an der Universitätsmedizin Rostock vom Studiendekan beauftragt, den der Verteilung von LOM Lehre zugrunde liegenden Kriterienkatalog zu überarbeiten. In diesem Zusammenhang wurde zunächst anhand von multiple choice-Klausurergebnissen ein Algorithmus entwickelt, der auf Aufgabenschwierigkeit, Trennschärfe, Reliabilität und Notenspiegel basiert und damit die Qualität der häufigsten Prüfungsform im Studium der Humanmedizin quantitativ abbildet.

**Ergebnisse: **Dieser Algorithmus wurde anschließend auch auf Klausuren mit offenen Fragen sowie auf den OSCE übertragen. Mit seiner Hilfe lässt sich die Prüfungsqualität in den einzelnen Fächern berechnen und – vergleichbar mit Impaktpunkten und Drittmitteleinwerbungen - in eine intrafakultäre Rangfolge überführen.

**Schlussfolgerung: **Dieser Algorithmus ist auf alle Prüfungsformate anwendbar, bei denen Aufgabenschwierigkeit, Trennschärfe, Reliabilität und Notenspiegel erfasst werden. Auch wenn eine weitere wichtige Kenngröße, nämlich die Validität einer Prüfung hier nicht berücksichtigt wird, so ist der vorgestellte Algorithmus als Bemessungsgrundlage für LOM durchaus geeignet.

## Introduction

„Assessment drives learning“. For the last 30 years, it has amply been analyzed and documented that we guide the learning styles and the academic performance of our students by the way we assess their knowledge [1], [2], [3], [4], [5]. In 2002, the amendment of the German Medical Licensing Act led to graded assessments in all clinical subjects and in an increasing number of interdisciplinary areas [http://www.gesetze-im-internet.de/_appro_2002/BJNR240500002.html]. As a general rule, these graded assessments are based on multiple choice (MC) tests.

This increase in graded assessments not only posed a logistic challenge for the faculties, but also offered the possibility to guide the students’ learning behavior and to create the conditions for improved performance in the second state exam. The latter though required that the faculty specific exams are of high quality. To help ensure this quality, the German Society for Medical Education (GMA) together with the German Association of Medical Faculties (MFT) published recommendations for the administration of high-quality assessments [6], [7]. These recommendations also provide quantifiable parameters like item difficulty and discrimination as well as the reliability of the exam as a whole. In general, the quantification of exam quality should be objective, reliable and valid. While objectivity and reliability can readily be quantified, validity can at best be estimated. 

To meet the logistic requirements for the many written exams, the medical faculty of Rostock in 2009 implemented an electronic item management system, the use of which is voluntary yet accepted by almost all clinical departments. Ever since there is transparency on the results of all exams and those responsible for the exams obtain detailed feed-back on passing-scores, distribution of scores and grades achieved, item difficulty and discriminatory power of each item. Nonetheless, little has changed for the faculty wide assessments and not meeting the quality standards did not necessarily lead to noticeable efforts in improving MC exam quality. In order to direct the faculty’s attention towards higher exam quality, we here decided to use exam quality as a criterion for calculating performance-based allocation of funds. However, in order to be accepted by the faculty and to lead to the desired effects, this calculation needed to be reproducible and transparent [8], [9]. Against this background, we here designed an algorithm to quantify exam quality as a basis on which to allocate performance based funds. 

## Methods

In the spring of 2014, the students‘ dean commissioned the „core group“ for curricular improvement at the University Medical Center in Rostock to revise the criteria for the allocation of performance-based funds for teaching. As a first step towards the integration of exam quality, we assessed already published parameters for high quality exams like item difficulty, discrimination and reliability [10], [11], [12]. However, to additionally meet the observed asymmetry in the grading of some departments, we also calculated any deviation from the Gaussian distribution. Based on the results of all exams written in the clinical subjects taught in the summer term of 2014, we developed an algorithm that would include all four parameters equally and would allow for a ranking of the results. The basis for these calculations was a matrix showing for all students which item was answered correctly (1) or incorrectly (0) and what score was reached, respectively. These matrices were either generated out of the electronic item management system or were compiled manually. Even though type A/5 options is the most common type of items used in our written exams, some departments within the faculty also use short assay questions and our fifth year is required to sit an OSCE.

In a first step, we calculated the proportion of items per exam or stations per OSCE that featured both, an item difficulty between 0.40 and 0.85 and a part-whole-corrected discrimination characterized by a Pearson correlation coefficient (r) of at least 0.2. Item difficulty was here defined as the percentages of students who had correctly answered an MC question of Type A or the mean scores of short assay questions or of OSCE stations, respectively. Chi-square tests to evaluate the distribution of grades achieved as well as Cronbach’s α were calculated in Excel. Subsequent correlation analyses performed with GraphPad InStat (Version 3) yielded Spearman-Rank correlation coefficients (r) and the corresponding 95%-confidence intervals (CI).

## Results

We here used item difficulty, discrimination, reliability and grade distribution as objectively measurable quality criteria for written tests. These four parameters were reduced to three by defining a proportion of “good” questions that showed both an item difficulty between 0.4 and 0.85 and a part-whole-corrected Pearson correlation coefficient (r) of at least 0.2. Parameters two and three were reliability (described as Cronbach’s α) and the distribution of the grades (described as the P value resulting from a chi-squared distribution test). 

**Figure 1:**
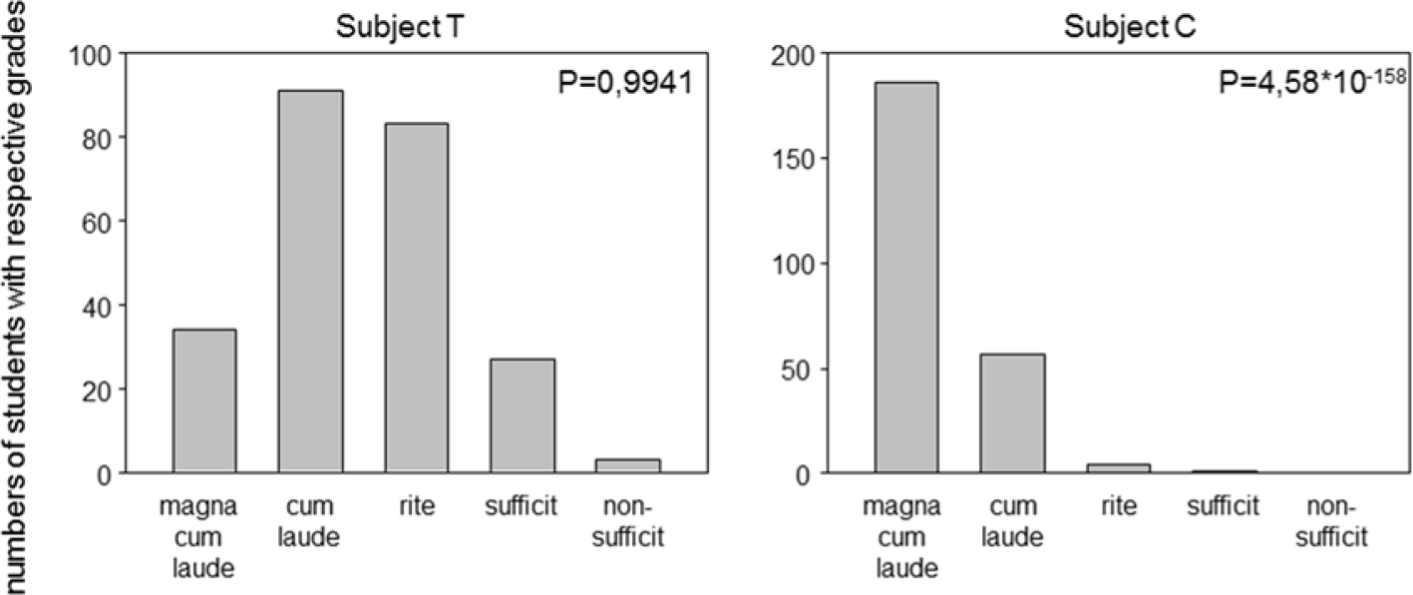
Figure 1: Extreme distributions of grades. Bar diagrams represent a Gaussian distribution of grades achieved for subjet T and a „ceiling effect“ for subject C (letters denote the same subjects as in Table 1).

The proportion of “good” questions ranged per definition between 0 and 1. A value of 1 resulted if all questions of an exam perfectly fulfilled both criteria, namely item difficulty and discrimination. Cronbach’s α can theoretically be negative however, evaluating written exams usually results in values between 0 and 1. As with the first parameter, “good” item, higher values represent better results. Chi-squared distribution of grades measures any deviation from the Gaussian distribution and resulting P-values smaller than 0.05 deny Gaussian distribution. In fact, the smaller the P-value, the more skewed the distribution of grades was. Evaluating all results obtained in the summer term in 2014 resulted in P-values between 4,6x10^-158^ and 0.99, respectively. Figure 1 (Fig. 1) presents two extreme grade distributions.

In order to weigh all three parameters equally, the resulting values describing the proportion of “good” questions, reliability (Cronbach’s α) and the distribution of grades (P-value resulting from the chi-squared distribution test) were transformed onto a scale between 0 and 1 with the highest values being 1 and the lowest being 0. Subsequently, the transformed values describing the three parameters were added up and the results were ranked (see Table 1 (Tab. 1)). Coefficients resulting from the ranks’ correlation of the proportion of “good” items, reliability and distribution of grades were 0.660, 0.1229 and 0.1225, respectively.

Table 1 (Tab. 1) summarizes the quality of 19 exams that were written at the University Medical Center of Rostock in the summer of 2014 and that were evaluated using the electronic item management system. These exams were MC featuring the item type A, only. Introducing exam quality as another criteria for the performance-based allocation of funds led to the manual compilation of matrices for those exams that were not yet managed via the electronic system so that item difficulty, discrimination, reliability and distribution of grades could eventually be evaluated for all exams. Thus, our algorithm was applied not only to exams including short assay questions but also to the OSCE.

## Discussion

Our algorithm presented here for evaluating exam and OSCE quality represents on the one hand internal consistencies – in the form of corrected discrimination and reliability – and on the other hand test results – in the form of item difficulty and grade distribution. Item difficulty, discrimination and reliability are already accepted as quality criteria in the medical literature [7], [13], [14], albeit we keep the lower limit of 0.4 for very difficult and the upper limit of 0.85 for very easy tasks also in short-assay tests and the OSCE. The lower limit could be re-defined in non-MC test formats, however, one has to take into consideration that a further lowering might impair discriminatory power. Likewise, the upper limit is debated in the sense that exams should also include questions that can be answered by each and every student. Here, each faculty needs to issue individual recommendations as to the maximum proportion of “easy” questions. 

The additional inclusion of grade distribution in our criteria is due to the observation that some departments consistently do not exploit the complete spectrum of possible grades (see Figure 1 (Fig. 1)). The resulting skewedness precludes internal differentiation and is in our opinion not suitable to support the learning behaviour of students [5]. With the algorithm presented here, we aim at receiving a normal distribution, in which we intentionally do not declare the middle grade (“rite”) as the mean, but allow for the individually calculated mean for each exam. We decided to assess the distribution of grades instead of scores achieved for two reasons,

1. there is no uniform maximum score in the written tests and
2. the scores achieved can potentially be normally distributed even if half of the students did not pass the exam.

Moreover, our algorithm is based on the P value which indicates the probability of deviation from the normal distribution, rather than the degree of deviation per se. We have decided to do so, because the P value and the 5% significance level are omnipresent and easy to understand. It remains to be considered that both the P value and the degree of deviation from the normal distribution depend on the sample size “N”. This, however, is not critical as long as student cohorts are of approximately same sizes and the number of participants in the exams per year are comparable. In the algorithm presented here, the proportion of „good” questions correlated with the reliability. This could be partly due to a redundancy of the criteria assessed, but it is also likely that a dedicated examiner would produce not only questions with high discriminatory power, but also will take into account the distributions of item difficulty and include more question items, which in turn will raise reliability. 

The algorithm presented here is a potential measure of good-quality exams. It can be applied for all test formats in which item difficulty, discrimination and grade distribution is recorded, and can therefore be used directly as a basis for performance-based funding. Once a transparent scoring system is established, item difficulty, discrimination and grade distribution can be calculated, even if the test formats are composite and performance is based on protocols or log books. 

In contrast to the students’ evaluation, which is most commonly used for the allocation of performance-based funding [9], the algorithm presented here has the advantage that exam quality is independent of the popularity of the subject. Nevertheless, this instrument also carries potential drawbacks: Subjects with high-quality written exams could insist on their reliable, but not necessarily valid test formats and thus prevent innovative changes. Here, the faculty could countersteer by not only assessing exam quality for performance-based funding, but also innovative teaching and learning formats. At the Rostock Medical School, performance-based funding for teaching consists of three criteria: exam quality, student evaluation and elective courses. Participation at the OSCE, an interdisciplinary event, is represented as an elective course where the quality of each station is used exclusively to guide the participating departments.

Whether indeed exam quality can be improved by the allocation of funds will only transpire after testing and evaluating this control instrument for several years. However, we are optimistic that the modified funding for teaching can at least draw more attention towards exams. A first objective, namely that those subjects who did not use the electronic exam management system before, now analyze their exams on qualitative criteria, has been achieved. The transparency of the applied criteria – item difficulty, discrimination and grades – offers the opportunity that teachers intensify reflection on their exams and seek to improve test quality [8], [15]. The algorithm presented here offers several possibilities for adjustment, among them reliability, that can most easily be influenced by the number of question items. In the past, a lack of discriminatory power has sporadically been used to review the distractors and to check the conformity of exam and course content. Ideally, the algorithm presented here will not only help to improve the quality of the individual question items, but will motivate the faculty members to question the validity of their tests, a parameter that cannot be assessed with our instrument. Ultimately, it remains to be seen whether and how an improved test quality will impact on the student evaluation on the one hand and on the performance in the second written state exam on the other hand. 

## Competing interests

The authors declare that they have no competing interests.

## Figures and Tables

**Table 1:**
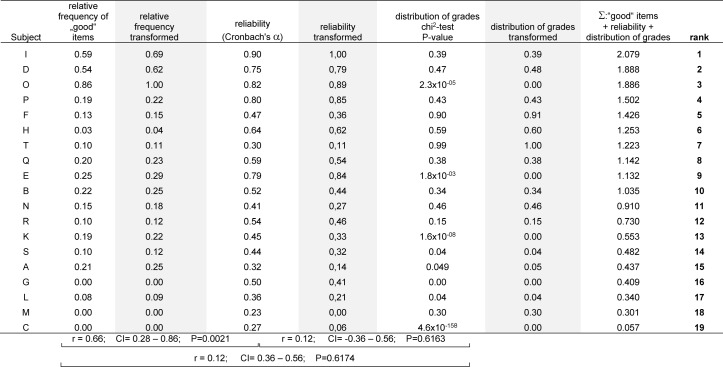
Algorithm for the calculation of exam quality
